# A Systematic Approach: Molecular Dynamics Study and Parametrisation of Gemini Type Cationic Surfactants

**DOI:** 10.3390/ijms222010939

**Published:** 2021-10-10

**Authors:** Mateusz Rzycki, Aleksandra Kaczorowska, Sebastian Kraszewski, Dominik Drabik

**Affiliations:** 1 Department of Experimental Physics, Faculty of Fundamental Problems of Technology, Wroclaw University of Science and Technology, 50-370 Wroclaw, Poland; 2 Department of Biomedical Engineering, Faculty of Fundamental Problems of Technology, Wroclaw University of Science and Technology, 50-370 Wroclaw, Poland; aleksandra.kaczorowska@pwr.edu.pl (A.K.); sebastian.kraszewski@pwr.edu.pl (S.K.); dominik.drabik@uwr.edu.pl (D.D.); 3 Laboratory of Cytobiochemistry, Faculty of Biotechnology, University of Wroclaw, 50-383 Wroclaw, Poland

**Keywords:** gemini, molecular dynamics, force field, parametrisation, antimicrobial, membranes

## Abstract

The spreading of antibiotic-resistant bacteria strains is one of the most serious problem in medicine to struggle nowadays. This triggered the development of alternative antimicrobial agents in recent years. One of such group is Gemini surfactants which are massively synthesised in various structural configurations to obtain the most effective antibacterial properties. Unfortunately, the comparison of antimicrobial effectiveness among different types of Gemini agents is unfeasible since various protocols for the determination of Minimum Inhibitory Concentration are used. In this work, we proposed alternative, computational, approach for such comparison. We designed a comprehensive database of 250 Gemini surfactants. Description of structure parameters, for instance spacer type and length, are included in the database. We parametrised modelled molecules to obtain force fields for the entire Gemini database. This was used to conduct in silico studies using the molecular dynamics to investigate the incorporation of these agents into model E. coli inner membrane system. We evaluated the effect of Gemini surfactants on structural, stress and mechanical parameters of the membrane after the agent incorporation. This enabled us to select four most likely membrane properties that could correspond to Gemini’s antimicrobial effect. Based on our results we selected several types of Gemini spacers which could demonstrate a particularly strong effect on the bacterial membranes.

## 1. Introduction

Antimicrobial resistance against available antibiotics has been acknowledged as one of the most serious problems in medicine nowadays. This resulted in a surge of new research works related to the synthesis of novel compounds that could serve as a potential modern-generation groups of antimicrobial particles. One of these groups are Gemini surfactants (initially referred to as bis-surfactants), which are heavily reported for their antimicrobial effect [1]. In recent years, Gemini surfactants have been heavily addressed in the world of science. Over the past five years, more than 130 articles dealing with the subject of Gemini surfactants have been published, among which researchers determined the methods of synthesis of new compounds, their physicochemical properties and even their potential use or application.

**Figure 1:**
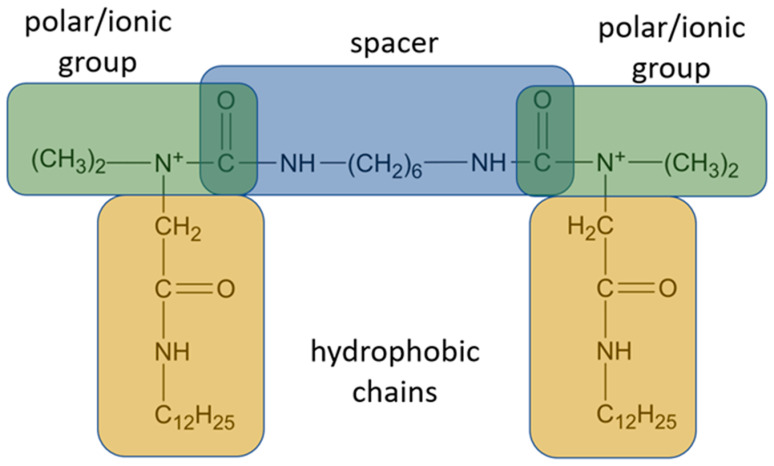
General scheme of Gemini structure classification presented on QAS-type spacer Gemini molecule.

Gemini surfactants have unique structural properties. They consist of two amphiphilic groups connected by a spacer at the head level, which can be both hydrophilic and hydrophobic [2,3]. They have at least two hydrophobic chains and two ionic or polar groups. There is a great variety in their structure e.g., short and long methylene groups can be used as a linker, stiff (stilbene), polar (polyether) and nonpolar (aliphatic) groups can be used as a linker [2,3]. The ionic group can be positive (ammonium) or negative (phosphorus, sulfur, carboxylase), while the polar non-ionic groups can be polyether or sugar. Most Gemini surfactants have a symmetrical structure with two identical polar groups and two identical chains (but there are also Gemini that are asymmetrical or with three polar groups or chains) [4]. A universal scheme of Gemini is presented in Figure 1.

A major part of synthesised Gemini surfactants has performed exquisite antibacterial properties against both Gram-positive and Gram-negative bacteria [5,6,7]. The most common antibacterial particles of this group are based on quaternary ammonium salts (QAS). Such salts prevent the development of bacteria and fungi; therefore, they are used on a large scale for cleaning, maintenance and disinfection. There were several attempts to evaluate the effectiveness of antibacterial activity of Gemini surfactants. However, these are usually limited to the compound structure—it is directly associated with the type and length of spacer in the molecule and/or the length of hydrophobic chains [8,9,10]. Numerous scientists proved that number of carbon atoms is correlated with the antimicrobial activity [11,12]. It has been established that a greater number of carbons in the molecule’s structure increases its antibacterial activity, and the presence of 12 carbon atoms cause the greatest antibacterial response. It was proposed that the shorter chains might not interact with the hydrophobic region of the bilayer as smoothly and immediately as the longer ones [13]. However, very long tails might curve and twist disqualifying the interactions with negatively charged membrane surfaces by covering cationic head groups. Although it is believed that the major element in the surfactant antimicrobial properties is connected to the hydrophobic chain. It was confirmed that the head group type and structure are also essential factors of biological activity as in the case of QAS molecules [14]. Moreover, Moran et al. revealed that the structure of the hydrophilic core also plays an important role in antimicrobial effects [15,16].

Nevertheless, as mentioned earlier, all works focus only on the structural differences of Gemini surfactants. Furthermore, the conclusions are usually limited to one subgroup of Gemini compounds, hence when analysed more globally, are often mutually contradictory. The reported antimicrobial activity is based on minimum inhibitory concentration (MIC), which strongly depends on the protocol used [17]. The studies reporting the interactions and the effect of Gemini on membranes—with particular emphasis on their properties and potential rupture—are scarce in the literature. There are only available few studies on commercially available Gemini surfactants such as octenidine (OCT) [18,19,20]. This is quite surprising as membrane destruction was emphasised as one of the potential targets for antimicrobial effect [21,22]. To this end, in our work we have focused on systematic theoretical studies of Gemini agents. Specifically, we have reviewed available literature and recreated the structure of the synthesised Gemini particle groups. This was followed by the classification of molecules into subgroups and the parametrisation of the compounds to create force fields for molecular dynamics studies. As a result, we obtained 250 valid force fields of Gemini class surfactants. Finally, we have selected valid representatives of the subgroups and investigated their interaction with lipid membranes. The selection of representatives was partially determined by the conclusions of structural studies. We ended with 25 selected particles used in molecular dynamics studies. The model membrane system was based on the inner membrane of *E. coli*. In this work we report the theoretical effect of Gemini class surfactants on properties and behaviour of the membrane. Additionally, the incorporation and behaviour of the molecules were also assessed. Based on our systematic characterisation of membrane system, we selected four parameters that were strongly affected by Gemini agents’ incorporation. Those were: area compressibility, bending rigidity, lateral diffusion coefficient and surface tension. This selection of impactful parameters allowed us to make a preliminary selection of Gemini molecules groups that could show strong antimicrobial effect from those analysed. This work could provide a means for more detailed studies of Gemini class surfactants and their interaction with lipid membrane models. Such systematic computational analysis provides in silico method to select, from the group of molecules, the ones that are most likely candidates for antimicrobial compounds. It can result in decreasing the amount of expensive synthesis work, which can restrain this type of studies. In a further perspective, it could help in initial scanning of the molecules and facilitate comparison between different MIC studies to determine valid candidates for next-generation antimicrobial substances.

## 2. Results and Discussion

### 2.1. Parametrisation

Variety of different Gemini type molecules are synthesised and characterised every year in various literature reports. However, usually their antimicrobial effectiveness is described by a single MIC experiment using various protocols and bacteria families, after which they are left forgotten. Perhaps the new antimicrobial agents, more effective than currently available, have been already synthesised. Due to the shortcomings of the MIC experiments and the inability of systematic comparison it could be impossible to use them. Furthermore, these molecules have a specific biophysical effect on membranes, although are rarely used in molecular dynamic studies due to the missing of an appropriate parametrisation. To this end, we have collected the structures of synthesised Gemini molecules from a significant number of recent literature reports [1,3,7,10,23,24,25,26,27,28,29,30,31,32,33,34,35,36,37,38,39,40,41,42,43,44,45,46,47,48,49]. Using SCIGRESS software, these structures were designed and preliminarily optimised in the water solvent. It was followed by their equilibration and determination of the Hessian matrix was carried out using Gaussian software. Finally, data from both geometry and a Hessian matrix were used for parametrisation of modelled particles and the force fields creation. This approach was successfully used beforehand to create force fields for various particles [18,50,51]. In Supporting Materials (SM) we have delivered the detailed base of modelled 250 particles (see Microsoft Excel datasheet) with optimised force fields (see included zip file). Force fields are ready-to-use in NAMD software however, a detailed description on how to prepare them for GROMACS users was also included. Molecules were divided into groups based on the origin of the spacer. Each molecule is characterised by the molecular scheme, segment name, spacer formula, length of the spacer, length and formula of chain components and the presence of organic salt. Additionally, based on the modelled molecule structure, partition coefficient (logP) and critical micelle concentration (CMC) were determined. Several molecules were presented as a preview in Table 1 while the total selection is included in Table S1. The theoretical value of logP could be useful for molecule selection as it can indicate whether the molecule incorporates into the membrane in the first place. On the other hand, CMC value may suggest the aggregation behaviour of investigated agents. However, it should be noted that the algorithm is based on phenomenological values hence CMC should be only considered as an approximation.

### 2.2. Membrane Characterisation

**Figure 2:**
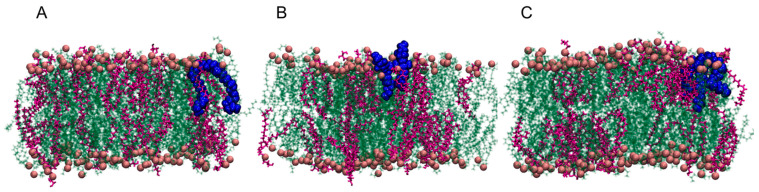
Selected screenshots of the systems with incorporated molecules, (**A**) Ole, (**B**) tQS, (**C**) hQS.

As previously stated, Gemini molecules are well-known for their antimicrobial activity. It was considered by Epand et al. [21] as well as shown in the OCT studies, that this effect is related to membrane disruption [18]. The effect of those Gemini molecules on the membrane properties was assessed to determine the properties that most likely correspond to the antimicrobial effect. A number of molecules were selected to investigate the effect of Gemini particles on membrane’s behaviour. Specifically, at least one molecule from each group was selected. Since in several works [52,53,54,55,56] it was reported that strongest antimicrobial effect was observed for Gemini agents with chain length equal to 12 carbon atoms, such condition was adapted during molecule selection from the group. All of the investigated molecules, except diGalactose (dGl), were incorporated into the membrane during the simulation time. The dGl molecule fluctuated over the bilayer surface, maintaining a 30 ± 4 Å distance from phosphorous atoms in lipid heads. The explanation of a lack of incorporation for dGl most likely lies in the negative logP of the molecule. A detailed location of system components such as lipid fragments or Gemini molecules has been presented in the partial density chart in Figure S1–S5 in SM. Selected screenshots of the systems with anchored molecules are presented in Figure 2. The membrane composition was selected in such a way to most accurately reflect the inner membrane of *E. coli* [21,57].

Membrane with incorporated Gemini molecules was thoroughly analysed to determine its properties such as area per lipid, membrane thickness, interdigitation, penetration depth, lateral diffusion, bending and tilt rigidities, area compressibility and surface tension. From such set of parameters four most likely candidates were chosen that could correspond to the antimicrobial effect. Those are membrane compressibility, bending rigidity, lateral diffusion and surface tension. The detailed characterisation of Gemini effect on the membrane, including all of the investigated parameters, is presented in SM Table S2. In this work, we additionally simulated the OCT molecule and we used it as a positive control. We assume that the effect on the membrane of these commercially available molecules could serve as a guidepost regarding desirable changes in selected properties. Since other molecules might indicate a much different mode of action therefore in our selection, we took into account the possible different mechanisms. Such a mechanism could induce a different magnitude of parameter change. As a result, we were also considering, in our selections, the extremum parameter changes, not only guided by the tendencies given by the effect of OCT. Concerning our analysis, we selected the four most changeable bilayer parameters reflecting membrane-agent interaction and potential antimicrobial activity. We deliver a total set of parameters in SM Table S2. The rest of the determined parameters were not selected due to insignificant differences between the analysed systems. Membrane thickness was, in general, determined to be between 39 and 41 Å. The difference of 2 Å between extreme particles with uncertainty equal to 1 Å was enough to exclude this parameter as an influential one. Similarly, tilt bending ranged from 9.9 up to 10.9 fold K_b_T with an uncertainty of 0.3 fold K_b_T. For APL, when the leaflet in which the Gemini agent incorporated is analysed, the values range between 58 and 62 Å^2^. Only two cases are extreme, which are 67.0 ± 3.3 Å^2^ for aryl bispiridine (Ary) and 64.8 ± 2.7 Å^2^ for glucose (Glu). Interestingly, both of those spacers were highlighted as possible antimicrobial based on analysis of other significant parameters.

**Figure 3:**
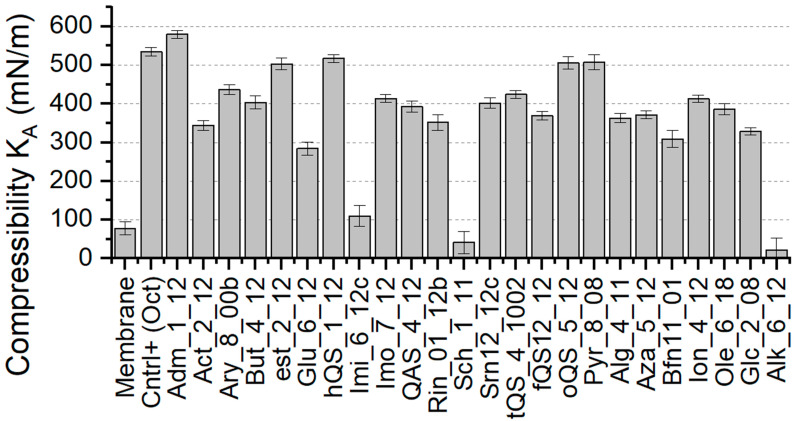
Determined values of compressibility (K_A_) for membrane system with incorporated Gemini molecules.

The area compressibility is one of the most robust parameters in our dataset, hence we predict that it may be an adequate property reflecting antimicrobial effect on the membrane. This mechanical parameter quantifies the energetic cost associated with the membrane’s area stretching and/or compressing. For high values of area compressibility, the membrane is resistant to external pressure. For low values the membrane loses its resistivity. Both cases can result in inability of proper cell function. The determined values of area compressibility of bilayers with incorporated Gemini molecules are presented in Figure 3. The area compressibility of positive control—membrane with incorporated OCT—is almost seven times higher than in the case of the model membrane. Interestingly, membrane area compressibility with incorporated Adamantane (Adm) is higher than in the case of positive control. Four other molecules from the ester (Est), higher quaternary ammonium salt (hQAS), oligomeric QAS (o-QAS) and pyridine (Pyr) group also induced significant growth in the area compressibility. Furthermore, two Gemini molecules had a decreasing effect on the area compressibility of the membrane. Specifically, those from Saccharide (Sch) and Alkyl Bispyridinamine (Alk) group.

**Figure 4:**
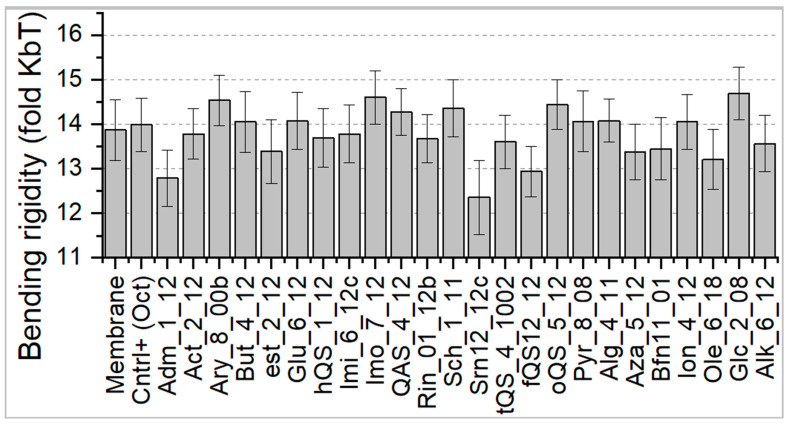
Calculated values of bending rigidity for membrane systems with incorporated Gemini molecules.

In our previous work we highlighted that mechanical parameter such as bending rigidity play an important role in OCT mode of action [18]. To this end we selected this parameter as a likely and promising candidate that correspond to antimicrobial activity. Briefly, bending rigidity quantifies the energetic cost associated with the membrane bending. The determined values of bending rigidity of membranes with incorporated Gemini agents are presented in Figure 4. First, the difference between pure membrane and the positive control is not statistically significant. To our knowledge, the OCT effect on bending rigidity is closely related to the aggregation properties rather than the effect of a single molecule action [18]. Nevertheless, several Gemini molecules significantly affected the bending behaviour of membranes. Considering OCT as a positive control we found the activity of several agents such as ionic (Ion), alginine (Alg), Pyr, butene (But) and glucose (Glu) as not statistically significant, hence similar to OCT. If, however, the strongest difference between pure membrane is considered, aryl bispiridine (Ary), imino (Imo), oQAS, gluconamid (Glc) were the most active candidates.

**Figure 5:**
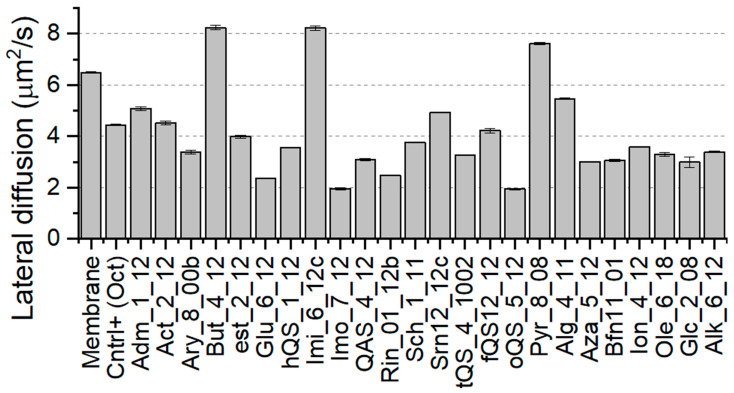
Determined values of lateral diffusion (2D) for membrane systems with incorporated Gemini molecules.

Lateral diffusion is a property that defines the mobility of lipid molecules in the membrane plane. Changes in lateral diffusion of the lipids could result in changes of movement of proteins, which can affect the activity of transporters and channel proteins [58]. Such an effect on proteins could significantly impede the functioning of microbe cells and correspond to antimicrobial effect of Gemini molecules. Although this dependency is not strictly related to the Gemini’s destructive effect on the membrane, it also should be considered as a factor. The values of lateral diffusion of lipid molecules are presented in Figure 5. Our positive control indicates that decreasing effect on lateral diffusion should be desirable however, the antimicrobial effect of OCT is based on membrane disruption and cannot be considered as a factor in this case. Results obtained for model membrane are in strong agreement with the GUVs *E. coli* mimicking studies, i.e., experimental diffusion coefficient equals D = 6.09 µm^2^/s [59]. Interestingly, different antimicrobial agent-thymol induced growth in lipids mobility, supporting the agent translocation [59]. To this end we selected three lowest and three highest values of lateral diffusion for selected agents that influence this membrane property. Gemini molecules that strongly increased the lateral diffusion of lipids on membranes were But, imidazolium (Imi) and Pyr. On the other hand, Gemini molecules that strongly decreased the lateral diffusion were o-QAS, Imo and Glu.

**Figure 6:**
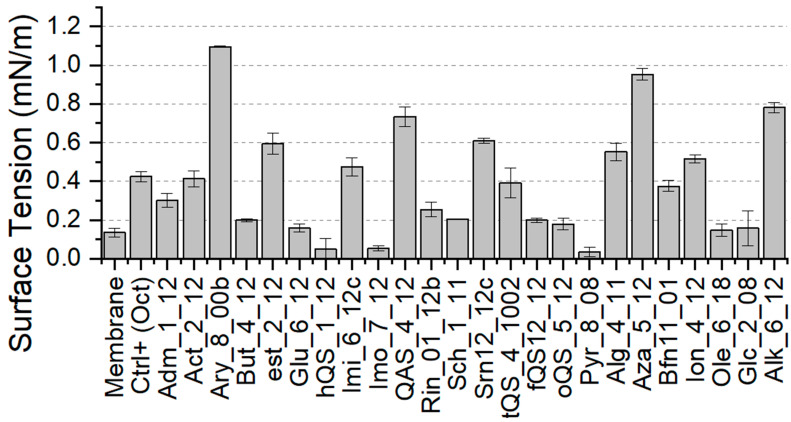
Surface tension, which was determined for membrane systems after the incorporation of investigated Gemini molecules.

Finally, the surface tension was determined for membranes with incorporated Gemini agents. Briefly, surface tension is defined as a cohesive force that keeps the cell membrane intact. Hence, its fluctuations may be very informative and extremely important for the determination of the antimicrobial mode of action based on the membrane disruption. Values of the membrane surface tension influenced by Gemini detergents are presented in Figure 6. The surface tension of membrane treated with OCT was twice as high than in the model membrane’s case. Interestingly, a significant number of investigated molecules had much stronger effect on membrane surface tension compared to the positive control. Three groups with the highest surface tension fluctuations were Ary, Aza and Alk. Additionally, the activity of several molecules such as hQS, Imo and Pyr led to decreased membrane tension. This should also be considered as change that could result in antimicrobial effect. Pure membrane exhibited natively certain surface tension hence, any strong deviation from this value could result in disruption of biological processes on the membrane.

Taking into account our results oQAS, Pyr, Imo groups from the Gemini family may exhibit strong antimicrobial effects. These molecules act as prominent candidates since three from four selected membrane parameters were significantly affected. Wang et al. in their experimental work [60] reported that presence of oxygen atom in oQAS spacer chain introduces higher flexibility and reduction of coulombic repulsion allows long side alkyl chain to tighter aggregation. This stays in line with our results since the oQAS molecules deeply penetrate the bilayer affecting membrane diffusion and mechanical properties. Moreover, Wettig et al. [61] highlighted unique transfection properties of Imo compared to other synthesised molecules since additional flexibility from extra methylene unit between nitrogen centres and readily protonated imino group is present. In our opinion, given molecule properties may influence the membrane resulting in limited tension and diffusion. Interestingly, both oQAS and Imo agents have similarities in structures (latter has additional nitrogen and methylene units in the spacer region) and induce comparable change in the membrane’s properties. Similarly, Quagliotto et al. [31] in the experimental work reported that increased Pyr concentration reduced the surface tension. This is in accordance with our theoretical approach where we observed significant limitations in membrane surface tension. Other vital candidates, that were selected based on two from four parameters, are Ary, Glu, hQAS and Alk. In the experimental work, Bailey et al. [1] concluded that Ary and Alk agents showed antimicrobial effectiveness, according to MIC. However, the latter showed weaker activity when compared to Ary. The authors emphasised that in the case of alkyl series the most effective agents are those with 22 up to 30 carbon atoms in the molecule. These could influence the character of membrane–molecule interactions and thus result in the fluctuation of membrane parameters. Our results also highlighted hQAS, which may be associated with molecule rigid spacer and three-charged headgroup indicating affinity to negative membranes [24]. Finally, Kumar et al. [38] reported that Glu shows excellent surface-active properties and low cytotoxicity, which stay in agreement with our findings based on membrane parameters variation. This selection was presented in Table 2. Despite suggested membrane thinning in reported experimental works we did not observe significant occurrence nor changes in acyl chain interdigitation in our studies (see Table S2) [62,63]. Moreover, in a significant part of analysed molecules, we observed their preferential localisation in the carbonyl-glycerol region. This was influenced by neither how long the alkyl chain nor the spacer were (see Figure S1–S5). Nevertheless, experimental comparison studies using uniformed protocol are required to confirm whether selected parameters directly correspond to the discussed antimicrobial effect.

## 3. Materials and Methods

### 3.1. Molecule Parametrisation

Quantum level calculations were performed using the Gaussian 2016 software package [64]. The equilibrium geometry of investigated Gemini molecules was calculated using density functional theory (DFT) (B3LYP)/6-31++G (d) level of theory; first with Loose Self Consistent Field (SCF) procedure, then with Tight. The solvent effect was taken into consideration using the integral equation formalism of the polarisable continuum model IEFPCM. Temperature was set to 300 K. Supplementary analysis based on the construction of the Hessian matrix (the matrix of second derivatives of the energy with respect to geometry) was also performed for further use in the force field parameterisation. The specific geometric and electronic data, such as bond lengths, angles, dihedrals and charge distribution were extracted from a Hessian matrix. The charge distribution was determined from the RESP charge calculations as being the most adapted to reproduce the molecular behaviour with the subsequently used CHARMM force field. For logP determination, the octanol/water partitioning coefficient was calculated using SCIGRESS software (SCIGRESS, Molecular modeling software, FQS Poland, ver. FJ-3.3.3). For CMC determination, the algorithm proposed by Mozrzymas was used [65]. It is based on phenomenological values and second-order connectivity index, that was determined using SCIGRESS software. Molecule schemes were prepared using MoleculeSketch (v. 2.2.3).

### 3.2. Molecular Dynamics Simulations

The all-atom models of the membranes were generated using CHARMM-GUI membrane builder [66]. The bacterial membrane model consisted of 80% PYPE, 15% PYPG, 5% PVCL2 [21,57]. The lipid bilayer was solvated with TIP3P water molecules (100 water molecules per lipid) and 240 mM NaCl were added based on literature data [67].

MD simulations were performed using the GROMACS (version 2020.4) package with the CHARMM36 force field [68,69]. Membrane systems were first minimised with the steepest descent algorithm for energy minimisation. Further calculations were carried out in the NPT ensemble (constant Number of particles, Pressure and Temperature) with Berendsen thermostat and barostat using semi-isotropic coupling at T = 303.15 K with time constant τ = 1 ps and p = 1 bar with τ = 5 ps. The primary part of the NPT calculations was performed using the leap-frog integrator with a 1 fs timestep. Afterwards, for the further NPT ensemble at T = 303.15 K, τ = 1 ps and p = 1 bar, τ = 5 ps, a Nose-Hoover thermostat [70] and Parrinello-Rahman barostat [71] were used. The second part of long-run production was carried out for 500 ns using the leap-frog integrator. Chemical bonds between hydrogen and heavy atoms were constrained to their equilibrium values with the LINCS algorithm, while long-range electrostatic forces were evaluated using the particle mesh Ewald (PME) method [72] with the integration timestep of 2 fs. Based on simulated pure membranes, the behaviour of Gemini surfactants was investigated. Molecules were placed on average 2.5 nm above the membrane leaflet and the same MD procedure was employed. For visualisation purpose, Visual Molecular Dynamics (VMD) was used [73].

### 3.3. Membrane System Characteristics

**Membrane Thickness and Area per Lipid.** Both area per lipid and membrane thickness were determined using self-made MATLAB scripts (Matlab R2019a). Briefly, for each leaflet Z-position on all phosphorus atoms were averaged, and distance between average Z-positions between each of leaflets was calculated for each frame. The final membrane thickness value is an average over analysed trajectory. Similarly, for each frame position of phosphorus atoms (or Gemini atom on the Z-level corresponding to phosphorus atoms) each leaflet was subjected to Voronoi tessellation. The average area for all lipid molecules was calculated for each leaflet and frame and was averaged over the analysed trajectory.

**Bending rigidity and Tilt rigidity.** Both bending rigidity and tilt rigidities were determined using self-made Matlab scripts that were based on the works of Doktorova et al. [74]. Briefly, a probability distribution for both tilt and splay are determined for all lipids over all analysed time steps. Tilt is defined as an angle between the lipid director (vector between lipid head–midpoint between C2 and P atoms–and lipid tail–midpoint between 16th carbon atoms) and bilayer normal. Lipid splay Sr is defined as divergence of an angle formed by the directors of neighbouring lipids providing that they are weakly correlated.

**Compressibility.** Compressibility was determined using self-made Matlab scripts based on the work of Doktorova et al. [75]. Briefly, a real-space analysis of local thickness fluctuations is sampled from the simulations for carbon atoms. This is followed by determination of reference surface and calculation of potential mean force from fluctuations from whole analysed trajectory to determine compressibility for given leaflet.

**Lateral Diffusion Coefficient.** The diffusion coefficient from the 2D mean square displacement (MSD) equation was calculated Diffusion Coefficient Tool [76] from the slope of the MSD curve through Einstein’s relation. This relation is presented in Equation (1), where M (t) is the MSD at a range of lag time tau and E represents the dimensionality (XY). For the computation accuracy, only phosphorous atoms (in the range of 20 Å from surfactant) of all lipids were considered.

(1) $$D\left(\tau \right)=\frac{M\left(\tau \right)}{2E\tau }$$

**Interdigitation.** For all provided systems the fluctuation of lipid interdigitation was determined using MEMBPLUGIN available in VMD software [77]. It is given in length units and reflects the interdigitation between opposite leaflets in the system—unless no interdigitation occurs it is equal to zero.

**Penetration Depth.** The depth of surfactant penetration was measured with respect to the membrane centre. From the last 50 ns of the trajectory the positions of the deepest placed carbon atoms on each alkyl chain were taken and evaluated with respect to the distance between phosphorous atoms divided by two, which represent the membrane centre.

**Surface tension.** The surface tension of membranes with anchored Gemini surfactants was computed using gmx energy function build-in GROMACS software using pressure tensor (Pxx, Pyy, Pzz values according to the Irving-Kirkwood method [78,79,80] and Equation (2), where L is the length of the simulation box in z dimension and represents an ensemble average given from gmx energy.

(2) $$\gamma =\frac{L}{2}\langle P_{zz}-\frac{P_{xx}+P_{yy}}{2}\rangle$$

**Significance test.** Significance tests were performed using OriginLab OriginPro 9.0 software. Specifically, one-way ANOVA was performed and was supplemented with post-hoc Tukey test to determine significance between individual populations.

## 4. Conclusions

In this work, we optimised and parametrised 250 Gemini molecules. We described each of those molecules with theoretical values of logP and log (CMC) as well as provided a detailed description of those molecules in the attached spreadsheet. Additionally, we included those parametrised force fields in SM for future simulation studies. This may be remarkably helpful in further antimicrobial action studies, as a significant number of Gemini cationic molecules with various spacers were modelled and parametrised. Such systematic summarisation may be extensively used not only for theoretical studies but also for experimental ones with the aim to deliver comprehensive knowledge and molecular mechanism of surfactant effectiveness. Furthermore, we selected 25 molecules from various groups and simulated their behaviour in systems with membrane mimicking the inner membrane of *E. coli*. This detailed characterisation of parameters allowed us to extract four types of parameters—area compressibility, bending rigidity, lateral diffusion coefficient and membrane surface tension—that could correspond to the antimicrobial effect of those molecules. Based on our preliminary screening we concluded that the type of Gemini molecules that could exhibit strong antimicrobial effects are oQAS, Pyr, Imo. Additionally, other possible candidates are Ary, Glu, hQAS and Alk. In this work we proposed and deliver a uniform theoretical approach to compare Gemini surfactant effectiveness. Nevertheless, this systematic approach should be confirmed experimentally to provide solid biological relevance.

## Figures and Tables

**Table 1:** Representation of selected modelled molecules from detailed base included in Supporting Materials.

Group	Scheme	ID	Seg Name	Linker	Linker Length (n)	Chain Compound (R1 or R2)	Number of Carbons in R1/R2 (m)	Number of Carbons from N+	Chemical Formula	Organic Salt	log10 (CMC)	Ref.
**Alkyl Bisp**	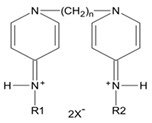	Alk_6_12	A6G	(CH_2_)_6_	6	C_12_H_25_	12	12	C_40_H_72_N_4_	Br	−4.09	[1]
**Aryl Bisp**	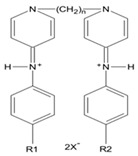	Ary_8_00b	AC2	(CH_2_)_8_	8	F	0	6	C_30_H_34_F_2_N_4_	Cl	−3.49	[1]
**fQAS**	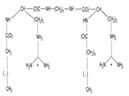	fQS12_12	F0A	C_12_H_16_F_8_	12	C_12_H_25_	12	12	C_40_H_78_F_8_N_2_	Br-	−6.65	[45]
**Ring**	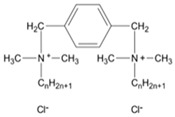	Rin_01_12b	RI1	C_6_H_4_	1	C_12_H_25_	12	12	C_36_H_70_N_2_	Cl	−3.63	[46]
**Ester**	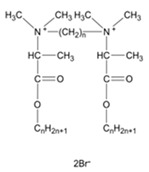	Est_2_12	E2D	C_2_H_4_	2	C_12_H_25_	12	15	C_36_H_74_N_2_O_4_	Br	−4.21	[35]
**Ionic**	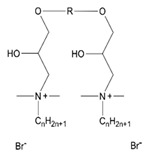	Ion_4_12	I4C	CH_2_CH_2_OCH_2_CH_2_	4	C_12_H_25_	12	12	C_38_H_82_N_2_O_5_	Br	−4.8	[10]
**QAS**	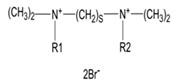	QAS_4_12	Q4A	C_4_H_6_	4	C_12_H_25_	12	12	C_32_H_70_N_2_	Br/Cl	−2.61	[27,36]
**tQAS**	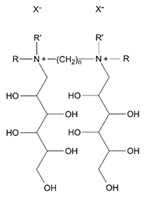	tQS_4_1002	T4F	(CH_2_)_4_	4	R1: C_10_H_21_ R2: C_2_H_5_	10 & 2	18	C_40_ H_86_ N_2_ O_10_	I	−5,82	[49]

**Table 2:** Selected potential antimicrobial candidates from each parameter group. It is suggested to compare those in experimental studies. Frequently appearing molecules were bolded to emphasise their repetition between different parameters consideration.

Compressibility K_A_ [mN/m]	Bending Rigidity [fold K_B_T]	Lateral Diffusion [µm^2^/s]	Surface Tension [mN/m]
**o-QAS**	**o-QAS**	**o-QAS**	Aza
**Pyr**	Ion	**Pyr**	**Pyr**
Adm	**Imo**	**Imo**	**Imo**
Est	**But**	**But**	
Sch	**Ary**	Imi	**Ary**
	**Glu**	**Glu**	
**hQAS**	Alg		**hQAS**
**Alk**	Glc		**Alk**

## Data Availability

Most of the data are available in the manuscript supplementary information, including force fields. The simulation data presented in this study are available on request from the corresponding author due to GBs files sizes.

## Supplementary Materials

The following are available online at https://www.mdpi.com/article/10.3390/ijms222010939/s1: (1) Gemini molecules database (.xls). (2) Complete force-fields for Gemini molecules from the database (.zip). (3) Supporting materials (.docx) including system density profiles of investigated agents, table with membrane system characterisation and instruction for NAMD FF to GROMACS FF conversion.
