# Supplementary material for: A Systematic Approach: Molecular Dynamics Study and Parametrisation of Gemini Type Cationic Surfactants
Source: Int J Mol Sci. 2021 Oct 10;22(20):10939. doi: 10.3390/ijms222010939 (PMC8536075; doi:10.3390/ijms222010939)
Supplement: Supplementary file 1 [file ijms-22-10939-s001.zip › Supporting_material_gemini_n.pdf]

A systematic approach: Molecular dynamics study and parametrization of Gemini type cationic surfactants

Mateusz Rzycki[1,2], Aleksandra Kaczorowska[2], Sebastian Kraszewski[2] and Dominik Drabik[2,3]

[1]Wroclaw University of Science and Technology, Faculty of Fundamental Problems of Technology, Department of Experimental Physics, Wroclaw, Poland

[2] Wroclaw University of Science and Technology, Faculty of Fundamental Problems of Technology, Department of Biomedical Engineering, Wroclaw, Poland

[3] Laboratory of Cytobiochemistry, Faculty of Biotechnology, University of Wroclaw, Wroclaw, Poland

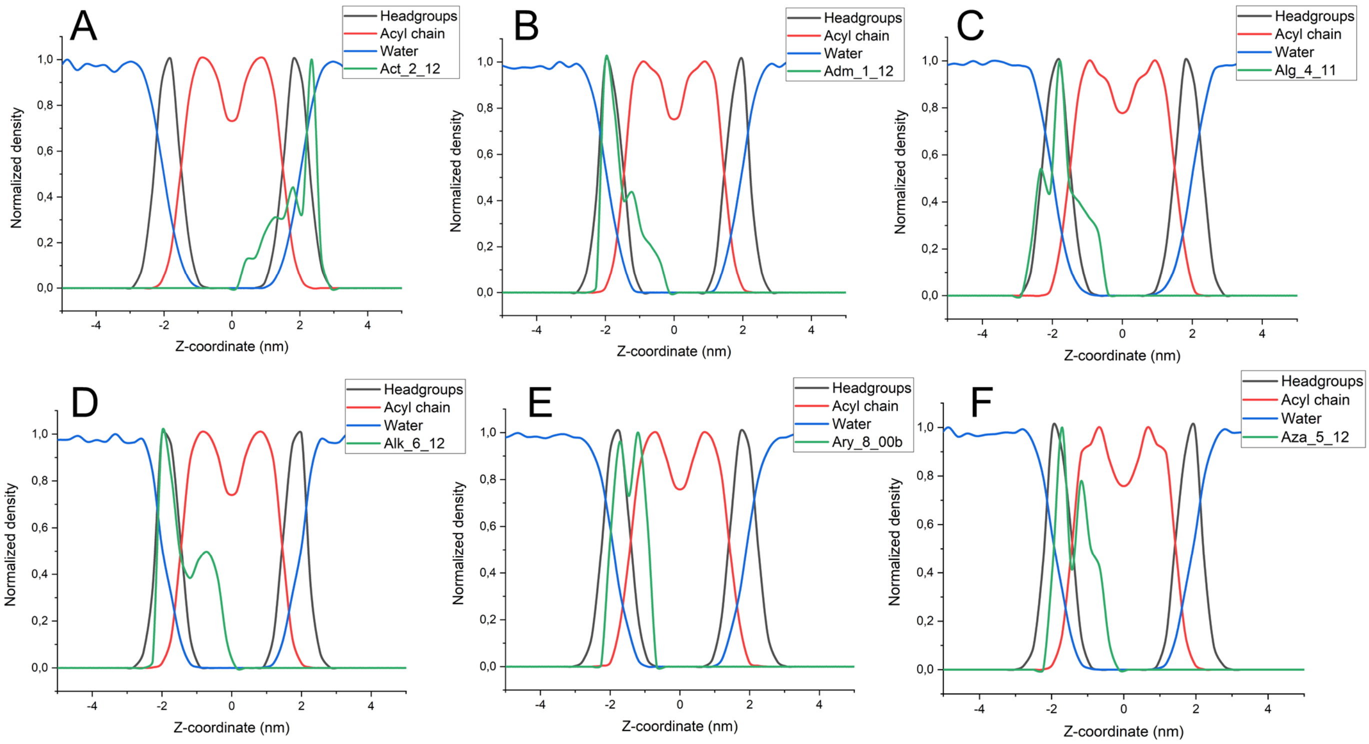

Figure S1 Density profiles of lipid bilayer regions and surfactant location along the axis normal to the membrane surface for (A) Act_2_12, (B) Adm_1_12, (C) Alg_4_11, (D) Alk_6_12, (E) Ary_8_00b, (F) Aza_5_12 Gemini agents.

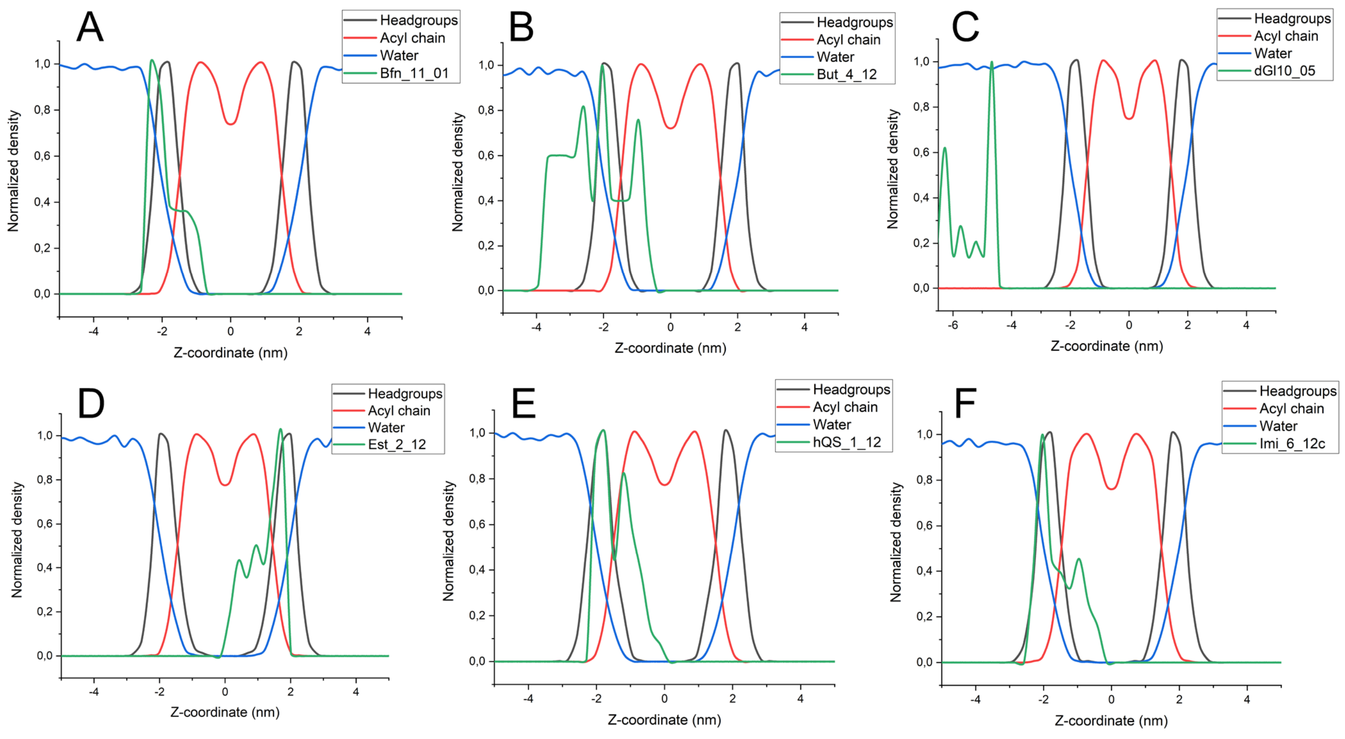

Figure S2 Density profiles of lipid bilayer regions and surfactant location along the axis normal to the membrane surface for (A) Bfn_11_01, (B) But_4_12, (C) dGl10_05, (D) Est_2_12, (E) hQS_1_12, (F) Imi_6_12c Gemini agents.

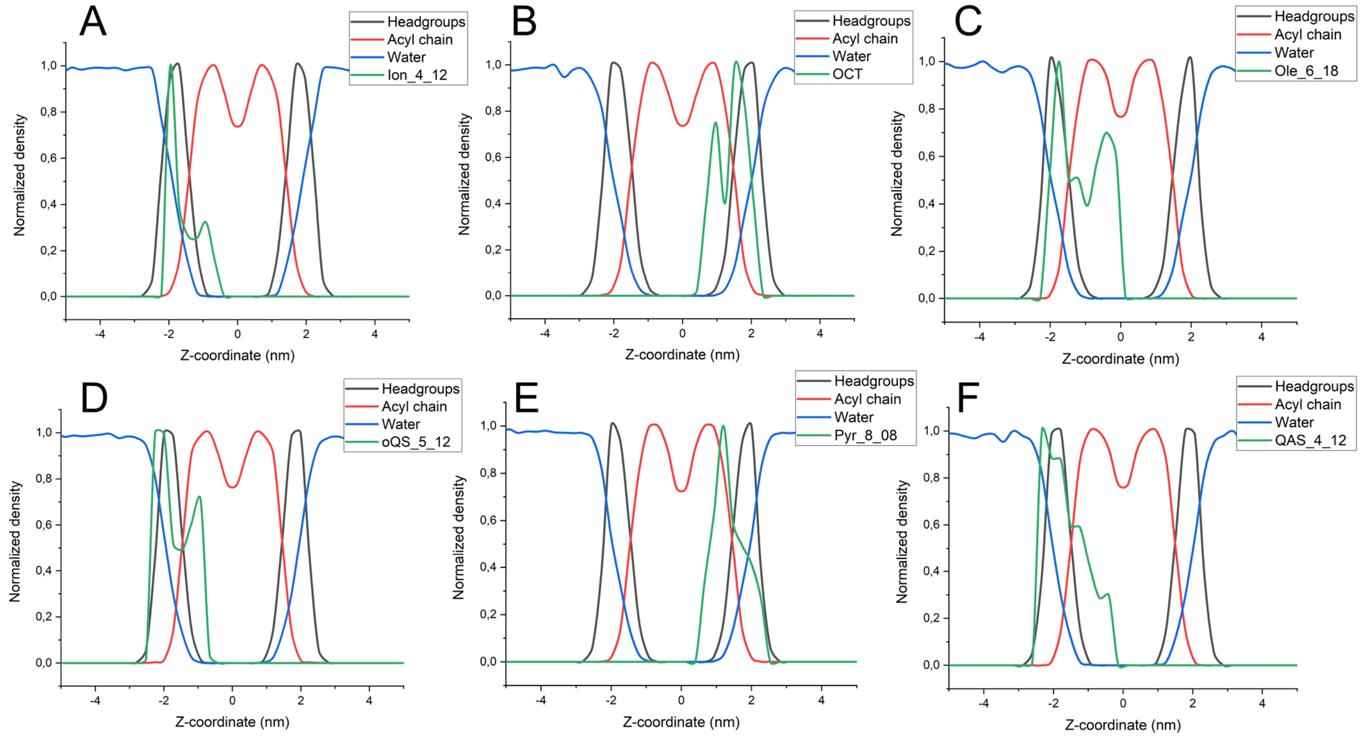

Figure S3 Density profiles of lipid bilayer regions and surfactant location along the axis normal to the membrane surface for (A) Ion_4_12, (B) OCT, (C) Ole_6_16, (D) oQS_5_12, (E) Pyr_8_08 (F) QAS_4_12 Gemini agents.

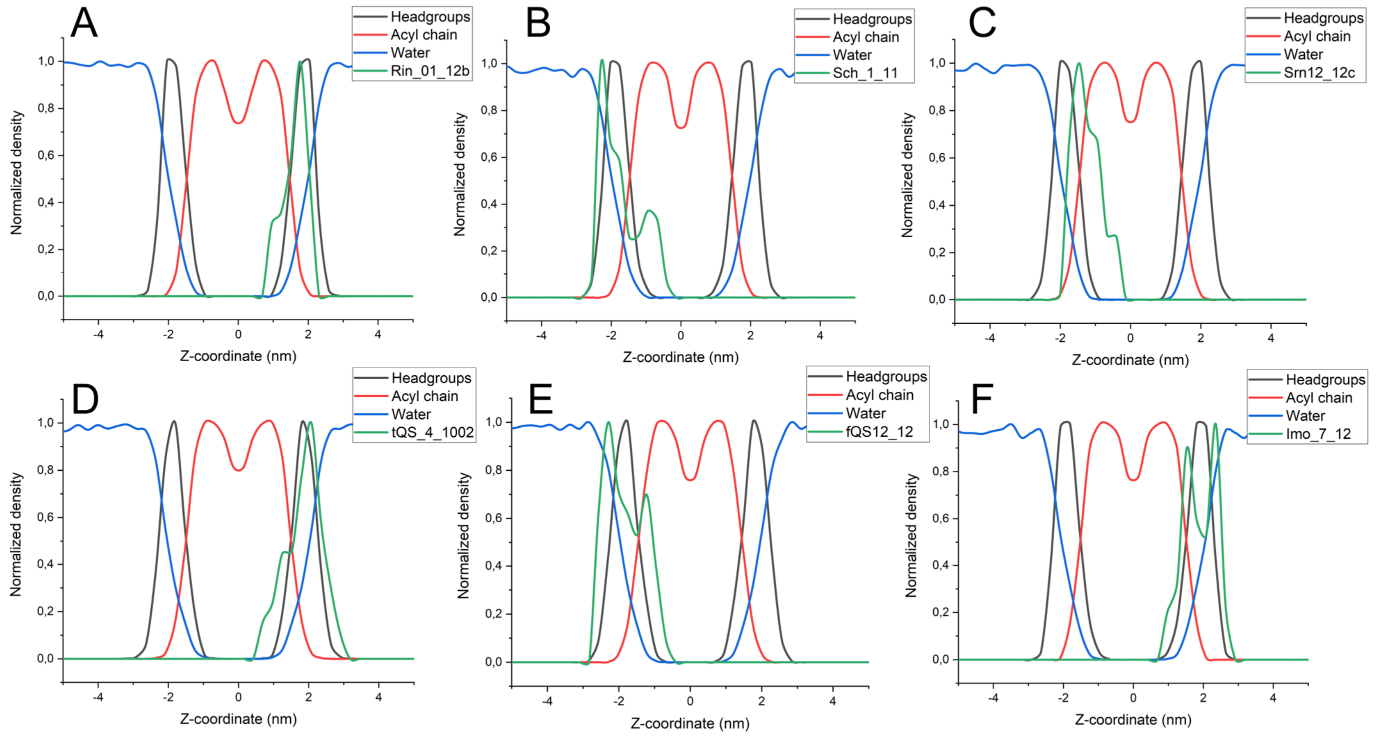

Figure S4 Density profiles of lipid bilayer regions and surfactant location along the axis normal to the membrane surface for (A) Rin_01_12b, (B) Sch_1_11, (C) Srn12_12c, (D) tQS_4_1002, (E) fQS12_12, (F) Imo_7_12 Gemini agents.

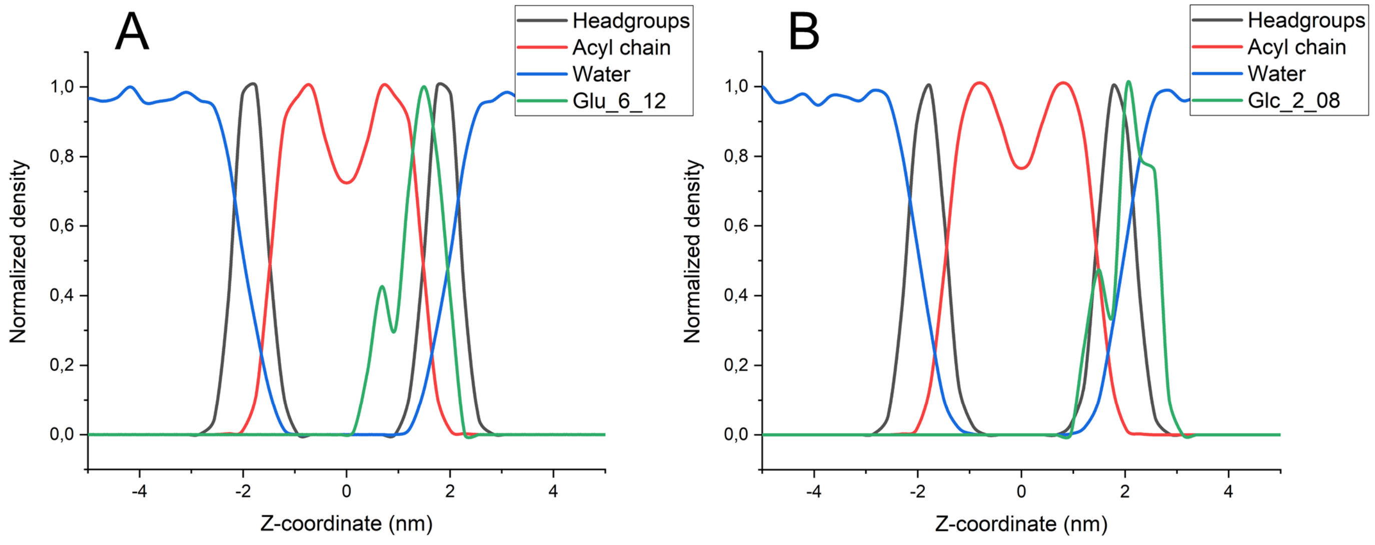

Figure S5Density profiles of lipid bilayer regions and surfactant location along the axis normal to the membrane surface for (A) Glu_6_12, (B) Glc_2_08 Gemini agent.

Table S1 Selected and modelled Gemini sufractants used in molecular dynamics studies.

Group	Scheme	ID	Seg name	Linker	Linker length (n)	Chain compound (R1 or R2)	Number of carbons in R1/R2 (m)	Number of carbons from N+	Chemical formula	Organic salt	log10(CMC)	Ref
**Alkyl Bisp**	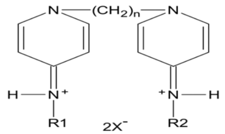	Alk_6_12	A6G	$(CH_2)_6$	6	$C_{12}H_{25}$	12	12	$C_{40}H_{72}N_4$	Br	-4,09	1
**Aryl Bisp**	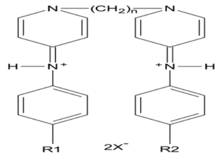	Ary_8_00b	AC2	$(CH_2)_8$	8	F	0	6	$C_{30}H_{34}F_2N_4$	Cl	-3,49	1
**Algine**	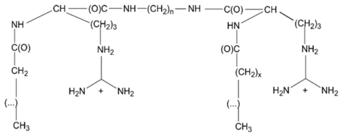	Alg_4_11	AL2	$(CH_2)_4$	4	$C_{11}H_{23}$	11	12	$C_{40}H_{82}N_{10}O_4$	Cl	-6,21	40
**Butene**	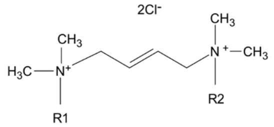	But_4_12	BU3	C_4H_6	4	$C_{12}H_{25}$	12	12	$C_{32}H_{68}N_2$	Cl	-3,3	37
**fQAS**	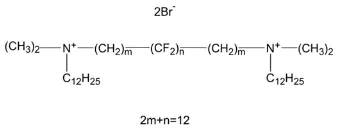	fQS12_12	F0A	$C_{12}H_{16}F_8$	12	$C_{12}H_{25}$	12	12	$C_{40}H_{78}F_8N_2$	Br-	-6,65	45
**Ring**	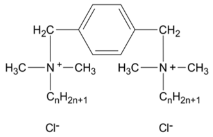	Rin_01_12b	RI1	C_6H_4	1	$C_{12}H_{25}$	12	12	$C_{36}H_{70}N_2$	Cl	-3,63	46
**Ester**	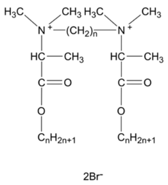	Est_2_12	E2D	C_2H_4	2	$C_{12}H_{25}$	12	15	$C_{36}H_{74}N_2O_4$	Br	-4,21	35

**Ionic**	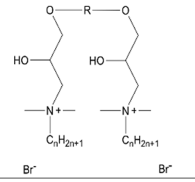	Ion_4_12	I4C	$CH_2CH_2OCH_2CH_2$	4	$C_{12}H_{25}$	12	12	$C_{38}H_{82}N_2O_5$	Br	-4,8	10
**QAS**	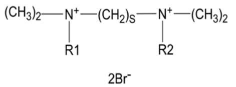	QAS_4_12	Q4A	C_4H_6	4	$C_{12}H_{25}$	12	12	$C_{32}H_{70}N_2$	Br / Cl	-2,61	27, 36
**tQAS**	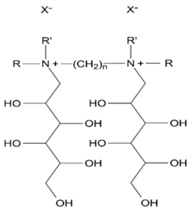	tQS_4_1002	T4F	$(CH_2)_4$	4	R1: $C_{10}H_{21}$ R2: C_2H_5	10 & 2	18	$C_{40}H_{86}N_2O_{10}$	I	-5,82	49
**Adamantane**	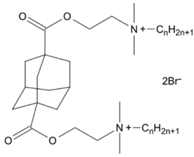	Adm_1_12	AD1	$C_{10}H_{16}$	1	$C_{12}H_{25}$	12	12	$C_{44}H_{84}N_2O_4$	Br	-6,57	41
**Acetyl**	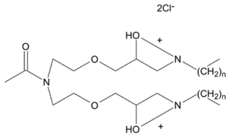	Act_2_12	AC2	NC_2OH_3	2	$C_{12}H_{25}$	12	12	$C_{40}H_{85}N_3O_5$	Cl	-5,55	42
**Glucose**	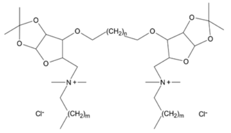	Glu_6_12	GL3	C_6H_{12}	6	$C_{12}H_{25}$	12	12	$C_{52}H_{102}N_2O_8$	Cl	-9,44	32
**hQAS**	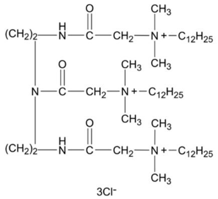	hQS_1_12	HQ1	N	1	$C_{12}H_{25}$	12	12	$C_{52}H_{109}N_6O_3$	Cl-	-8,16	45
**Imidazolium**	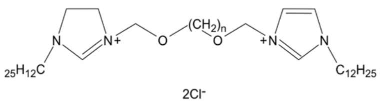	Imi_6_12c	M6B	$CH_2O-(CH_2)_4-CH_2O$	6	CH_2	12	15	$C_{38}H_{72}N_4O_2$	Cl-	-4,1	28

**Imino**	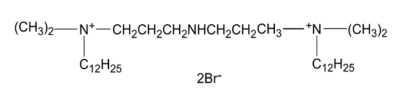	Imo_7_12	IMO	C_7NH	7	$C_{12}H_{25}$	12	12	$C_{34}H_{75}N_3$	Br-	-3,13	45
**Sacharide**	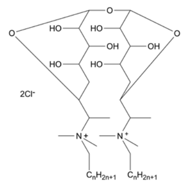	Sch_1_11	SA1	O	1	$C_{11}H_{23}$	11	11	$C_{44}H_{90}N_2O_9$	Cl	-7,34	7
**Serine**	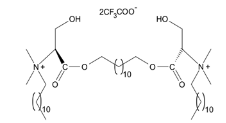	Srn12_12c	SE3	$C_{12}H_{24}$	12	$C_{12}H_{25}$	12	12	$C_{46}H_{94}N_2O_6$	CF_3COO	-6,17	44
**O-QAS**	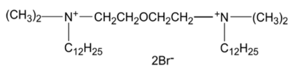	oQS_5_12	OQS	$(CH_2)_2O(CH_2)_2$	5	$C_{12}H_{25}$	12	12	$C_{32}H_{70}N_2O_1$	Br-	-2,78	45
**Pyridine**	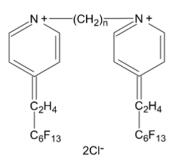	Pyr_8_08	PY3	C_8H_{16}	8	$C_8H_4F_{13}$	8	13	$C_{34}H_{32}F_{26}N_2$	Cl	-11,2	25
**Aza**	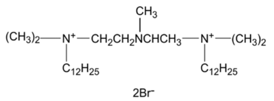	Aza_5_12	AZ5	C_5N	5	$C_{12}H_{25}$	12	12	$C_{33}H_{73}N_3$	Br-	-3,09	45
**Bifunction**	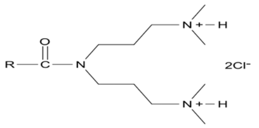	Bfn11_01	BF1	$N\text{-}CO\text{-}C_{11}H_{23}$	11	C_5NH_{13}	1	4	$C_{22}H_{49}N_3O_1$	Cl-	-0,94	38
**Oleyl**	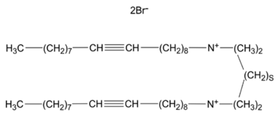	Ole_6_18	OL6	C_6H_{12}	6	$C_{18}H_{37}$:1	18	18	$C_{46}H_{94}N_2$	Br-	-5,04	45
**Gluconamid**	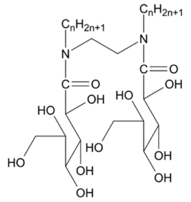	Glc_2_08	GU1	C_2H_4	2	C_8H_{17}	8		$C_{30}H_{60}N_2O_{12}$	N/A	-4,3	33

Table S2 The effect of Gemini agents' incorporation on model membrane structural and dynamic parameters.

Name	Membrane thickness	Area per lipid (other leaflet)	Area per lipid (Gemini leaflet)	Bending rigidity	Tilt	Compressibility	Interdigitation	Penetration depth	Diffusion coefficient	Surface tension
	Å	$Å^2$	$Å^2$	fold K_bT	fold K_bT	$mN*m^{-1}$	Å	Å	μm^2*s^{-1}	$mN*m^{-1}$
**Membrane**	39.7±0.5	58.4±2.1	58.66±2.12	13.9±0.7	10.5±0.3	78±17	4.7±0.5	-	6.49±0.02	0.13±0.02
**Ctrl+ (OCT)**	39.71±1.04	59.2±2.6	60.53±2.64	14.0±0.6	10.5±0.3	534±11	4.05±0.93	10.5±2.4	4.44±0.03	0.42±0.03
**Adm_1_12**	39.4±1.1	60.21±3.5	59.3±2.6	12.79±0.63	10.7±0.3	579±10	4.01±0.76	15.9±2.2	5.08±0.06	0.30±0.04
**Act_2_12**	39.5±1.1	62.0±2.5	59.1±2.4	13.8±0.6	10.58±0.25	344±13	4.56±0.64	5.4±2.2	4.53±0.07	0.41±0.04
**Ary_8_00b**	39.6±1.0	60.8±2.8	67.0±3.3	14.5±0.6	10.0±0.3	436±13	4.57±0.64	14.6±2.0	3.39±0.08	1.09±0.01
**But_4_12**	39.5±1.1	61.6±2.6	59.65±2.73	14.0±0.7	10.56±0.26	403±17	4.53±0.67	5.5±2.3	8.24±0.09	0.20±0.01
**Est_2_12**	40.7±0.8	59.43±2.34	60.7±2.1	13.4±0.7	10.7±0.3	502.9±15.1	4.67±0.56	5.9±2.4	3.99±0.06	0.59±0.06
**Glu_6_12**	39.59±1.31	62.2±3.0	64.78±2.67	14.07±0.64	10.34±0.36	283.7±16.5	4.10±0.83	6.5±2.9	2.37±0.02	0.16±0.06
**hQS_1_12**	40.2±1.2	59.3±2.9	62.49±3.21	13.7±0.7	10.85±0.32	517.0±10.3	4.32±0.60	5.7±2.1	3.56±0.01	0.05±0.05
**Imi_6_12c**	39.75±1.04	60.8±3.5	62.1±2.7	13.78±0.65	10.2±0.3	110±27	4.6±0.7	6.02±1.94	8.22±0.08	0.47±0.05
**Imo_7_12**	39.2±1.0	61.7±3.0	59.5±2.4	14.6±0.6	10.4±0.3	413.5±10.6	4.43±0.54	13.0±2.3	1.96±0.04	0.05±0.01
**QAS_4_12**	39.3±1.0	61.3±2.6	61.6±2.8	14.27±0.52	9.94±0.24	392±40	4.4±0.8	7.28±2.04	3.10±0.04	0.73±0.05
**Rin_01_12b**	39.51±1.03	60.9±2.8	60.8±2.4	13.68±0.54	10.3±0.3	351±20	4.5±0.8	8.6±2.1	2.48±0.02	0.25±0.04
**Sch_1_11**	39.8±0.9	63.9±3.1	60.41±2.24	14.35±0.64	10.39±0.26	41±29	4.4±0.6	5.6±1.8	3.76±0.01	0.20±0.01
**Srn12_12c**	39.0±0.9	60.6±2.5	63.3±3.2	12.36±0.83	10.2±0.3	401.5±13.7	4.6±0.6	8.1±2.8	4.93±0.01	0.61±0.01
**tQS_4_1002**	39.51±1.12	61.87±2.71	59.6±2.8	13.6±0.6	10.6±0.3	423.8±10.4	4.3±0.8	7.06±1.74	3.28±0.01	0.39±0.08
**fQS12_12**	39.4±1.0	60.89±2.34	60.7±2.9	12.9±0.6	10.3±0.3	368±11	4.6±0.6	8.66±2.23	4.22±0.08	0.20±0.01
**oQS_5_12**	40.0±1.0	61.65±2.44	59.45±2.72	14.4±0.6	10.46±0.32	505.4±16.1	4.51±0.64	6.9±1.9	1.95±0.03	0.18±0.03
**Pyr_8_08**	39.6±0.9	62.2±2.2	60.3±3.0	14.0±0.7	10.29±0.26	507.4±20.2	4.8±0.6	7.92±2.54	7.62±0.04	0.03±0.03
**Alg_4_11**	39.9±0.9	60.0±3.1	61.45±2.54	14.1±0.5	10.2±0.3	362±12	4.4±0.6	7.8±2.0	5.47±0.02	0.55±0.04
**Aza_5_12**	39.5±0.8	60.9±3.4	61.69±3.42	13.38±0.63	10.3±0.3	371.5±10.3	4.8±0.7	7.2±2.3	3.01±0.01	0.95±0.03
**Bfn11_01**	40.1±1.0	63.8±2.8	60.20±2.53	13.5±0.7	10.3±0.3	309.0±21.4	4.2±0.9	20.4±2.2	3.06±0.03	0.37±0.03
**Ion_4_12**	39.4±0.9	63.2±3.0	60.6±2.8	14.05±0.61	10.6±0.3	412.9±9.2	4.51±0.53	7.9±2.1	3.59±0.02	0.52±0.02
**Ole_6_18**	39.7±0.8	57.9±2.8	61.14±2.64	13.2±0.7	10.5±0.3	386±15	4.41±0.65	4.7±3.8	3.30±0.07	0.15±0.03
**Glc_2_08**	40.0±1.1	61.2±2.6	61.5±2.53	14.7±0.6	10.2±0.3	328.2±9.7	4.45±0.61	11.5±2.5	2.99±0.2	0.16±0.09
**Alk_6_12**	39.4±1.0	62.22±3.04	58.87±3.34	13.57±0.63	10.4±0.3	22.6±29.4	4.41±0.54	5.4±2.2	3.39±0.02	0.78±0.03

File preparation for GROMACS users

In this work, we prepared a database with a significant number of ready-to-use Gemini surfactants structures and topologies. Provided files are intended for NAMD software, however may be easily translated to fit GROMACS users. On that purpose we recommend using Python 2 tool made by Reza Salari called *PyTopol: A Library For Converting Molecular Topologies*. Further, we present a brief tutorial on how to deal with delivered NAMD force fields.

Firstly, ensure that you have Python 2.7 installed. You can clone the git repo or simply download it from the Reza Salari GitHub page https://github.com/resal81/PyTopol/wiki/PyTopol-Installation or use the pip installer. Afterward, copy desired topology files into the main PyTopol directory and use the following command to generate .top and .itp files.

```
psf2top.py -p psf_file.psf -c parameter_file.par
```

Further, comment redundant and overlapping headings in the .top file if needed. (in general first three lines, otherwise GROMACS will prompt when calling *gmx grompp* function). All the details and test section are available on the GitHub page.
